# Healthcare Provider's Preferences on Open Versus Restricted Visiting Hours in Surgical Intensive Care Unit

**DOI:** 10.7759/cureus.69871

**Published:** 2024-09-21

**Authors:** Raquel E Candal, Piyush Kalakoti, Beatriz Briones, Jane G Sugar, Terry C Lairmore, Robert Keith White, Navdeep S Samra

**Affiliations:** 1 Surgery, Louisiana State University Health Sciences Center Shreveport (LSU Health Shreveport), Shreveport, USA; 2 Surgery, Yale School of Medicine, New Haven, USA; 3 Public Health, Johns Hopkins Bloomberg School of Public Health, Baltimore, USA; 4 Trauma and Acute Care Surgery, Louisiana State University Health Sciences Center Shreveport (LSU Health Shreveport), Shreveport, USA

**Keywords:** burnout, healthcare survey, icu, intensive care unit, physician burnout, provider burnout, visiting hours

## Abstract

Background: The increasing consideration of open visiting hours in intensive care units (ICUs) has presented implementation challenges. Recognizing the invaluable insights of healthcare providers, this study aimed to assess their perspectives on the potential impact of altering ICU visiting policies on patient care.

Materials and methods: In a single-institution, cross-sectional study, a five-point Likert survey was administered to healthcare providers working in a surgical ICU to assess their perceptions of open and restricted visiting hours. Descriptive statistics were used to analyze the responses.

Results: Ninety-three healthcare providers participated, including nurses (53%), resident physicians (23%), and other healthcare professionals. The majority (81%) did not believe that open ICU visiting hours would enhance patient-centered care. Providers expressed concerns that open visitation could lead to increased provider stress (89%), physical barriers to patient access (80%), disruption in workflow (90%), and provider burnout (76%). Conversely, they perceived that restricted visitation would preserve patient privacy (87%), promote better healing for patients (64%), reduce provider stress (81%), and improve workflow (95%). Nurses were more likely than resident physicians to perceive that open ICU hours would alleviate familial stress (p=0.022). Additionally, both nurses and other healthcare providers recognized the potential for open ICU hours to improve communication between patient families and healthcare teams (p<0.05). Notably, there were no significant differences among providers in perceptions of the impact of open versus restricted visitation on provider stress, workflow, or other factors (p>0.05).

Conclusions: This survey reveals a negative perception of open ICU visiting hours among healthcare providers in a surgical ICU, with concerns that such a change could increase stress for both patients and providers. These findings underscore the potential challenges and negative impacts of implementing open visiting hours in this setting, emphasizing the need for careful consideration and further research to inform policy decisions and visitation practices.

## Introduction

Intensive care unit (ICU) visitation policies are gradually shifting towards open visiting hours. Also referred to as "extended," "flexible," or "unrestricted" visiting hours, an open visitation model limits restrictions on the time or length of visits and the number of visitors [1]. While traditional restrictive policies remain prevalent in most ICUs, adopting flexible visitation in select healthcare settings has prompted a critical evaluation of its impact on patient care and outcomes.

Proponents of open visitation emphasize its potential to enhance patient-reported outcomes (PROs) and facilitate family-centered care [2-4], including stress reduction and improved family satisfaction [5]. A growing body of evidence suggests that flexible visiting hours may alleviate delirium and anxiety in ICU patients without adversely affecting mortality rates, acquired infections, or length of stay [6-10]. However, these findings are not universally consistent, with some studies reporting conflicting results regarding the benefits of open visitation [11,12]. Furthermore, implementing an open visitation model presents unique challenges, including potential disruptions to patient care routines, disorganization of care delivery [5], risks of infectious complications [13,14], and increased provider burnout [15]. Notably, most previous research on open visitation has been conducted in medical or non-surgical ICUs, leaving a significant knowledge gap regarding its effects on surgical ICUs, which cater to acutely ill and trauma patients. Moreover, many of the published studies originate from non-US centers [5,9,11,12,16-20], limiting the generalizability of their findings.

In the United States, the lack of standardized ICU visitation practices further complicates the assessment of healthcare professionals and families' experiences and perspectives [15,21]. Existing research depicts substantial variability in visitation policies across different ICUs, hindering the ability to draw definitive conclusions [22]. This study addresses these knowledge gaps by surveying healthcare providers' perceptions of the impact of open vs. restrictive ICU visitation models on patient care. Focusing on the surgical ICU setting and gathering data from US providers, this research seeks to contribute valuable insights to the ongoing discussion regarding the optimal approach to ICU visitation and underscores the need for further research in this area.

A portion of the study was presented at the Annual Academic Surgical Congress in Orlando, Florida, on February 6, 2020.

## Materials and methods

The Institutional Review Board of Louisiana State University Health Sciences Center Shreveport (LSU Health Shreveport) approved the study (approval number: 00001204).

Study design and setting

A cross-sectional study was designed to survey healthcare providers at our surgical ICU, which serves patients from the level 1 trauma center in the Arkansas-Texas-Louisiana (Ark-Tex-La) region of the Southern United States. Our tertiary healthcare system follows a restrictive ICU visitation policy, allowing families to visit patients during four predefined 30-minute intervals, totaling two hours daily. To improve patient safety and outcomes as part of our ongoing quality initiatives, we recognized the growing national debate on open versus restricted visitation policies. Given that healthcare providers are key stakeholders in patient care and the cornerstone of healthcare delivery, we sought to evaluate their perceptions on this issue, which formed the basis for this study.

Study participants and workflow

In the spring of 2019, a three-part survey questionnaire was developed and pre-tested with input from surgical and critical care attendings. This collaborative effort was led by the Department of Surgery, Division of Trauma and Acute Care Surgery. The survey aimed to capture healthcare providers' perceptions of open versus restrictive visitation policies. Before the questionnaire, the survey included a preface that provided context on the increasing trend of open visiting hours in ICUs and highlighted the lack of understanding regarding its impact in surgical ICUs. It briefly outlined the pros and cons of the open visitation model and the study's overall goal: to gather healthcare providers' perspectives on how different visitation policies might influence patient care. The questionnaire was designed to collect data on three key areas: (I) respondents' demographics, (II) their perceptions of open and restrictive visitation policies, and (III) open-ended responses. Part II of the survey featured five-point Likert-scale questions. A copy of the survey is available in the Appendices. The term "open visiting" hours was deliberately left undefined to allow for open interpretation, considering the variability in the literature on open versus restrictive policies and the lack of standardized terminology.

Between March and May 2019, the survey was electronically administered to 200 healthcare providers in our surgical ICU, including surgical residents, residents rotating through the surgical ICU, mid-level providers, nurses, and allied health professionals. Surgical ICU attendings were excluded as they served as study coordinators.

Outcomes

The primary outcome measure assessed provider beliefs regarding the open and restricted visitation models in the surgical ICU setting on a five-point Likert scale: strongly agree, agree, neutral, disagree, and strongly disagree. Questions focused on preferences and perceptions of open versus restricted visitation, with response options ranging from "strongly disagree" to "strongly agree." The questions were centered on changing the current practice of restricted visiting hours to open visiting hours.

Statistical analysis

Responses from all professions on a five-point Likert scale were pooled, and descriptive statistics were calculated. F-tests using one-way analysis of variance (ANOVA) were conducted to compare the mean responses across healthcare providers. Tukey's Honestly Significant Difference (HSD) test was performed following a significant ANOVA result to identify specific pairwise differences between professions. All statistical inferences were made with a derived type I error rate set at 5% (α=0.05).

## Results

**Figure 1:**
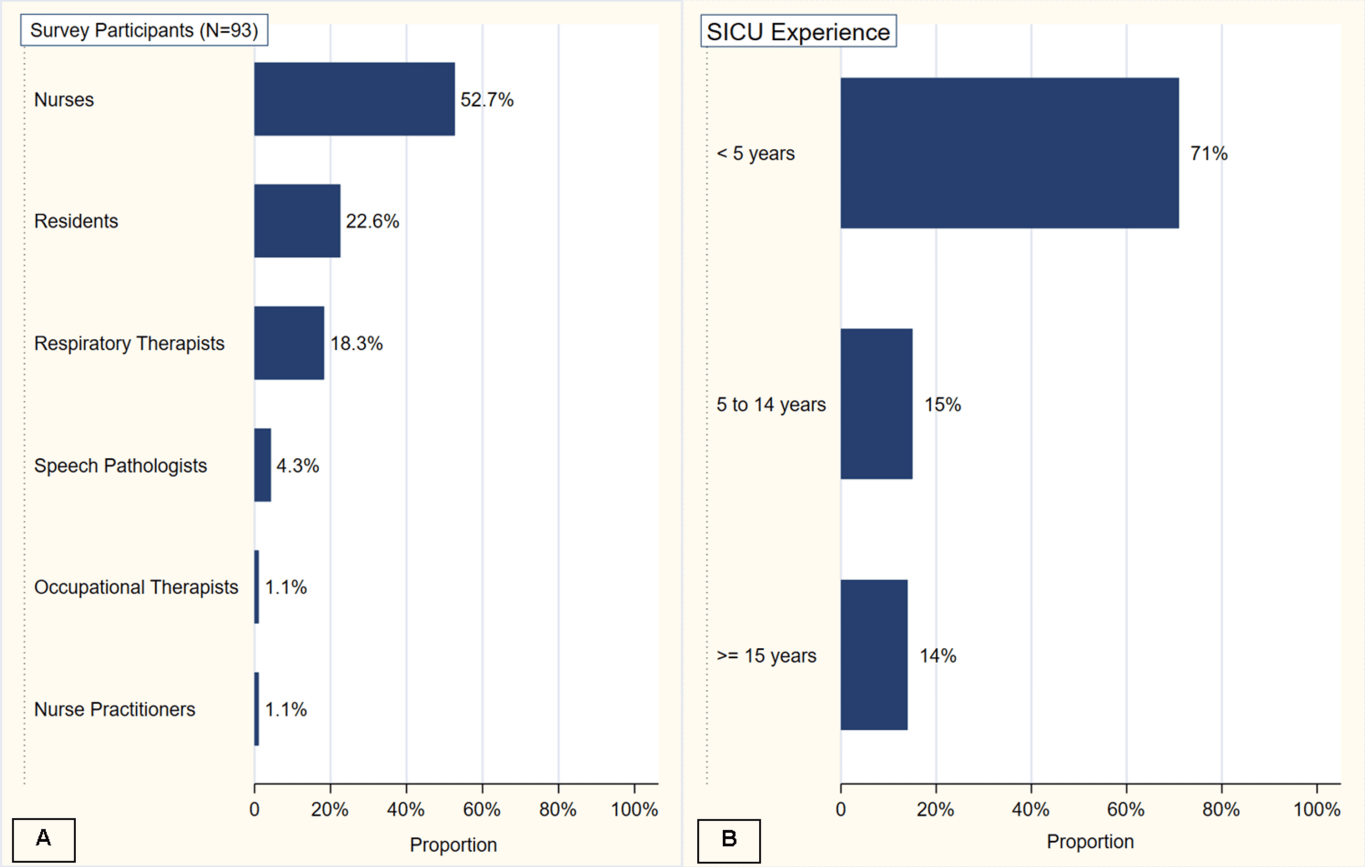
Descriptive summary of healthcare providers based on occupation and years of experience in SICU (A) Proportion of respondents based on their healthcare profession (B) Proportion of respondents based on their experience in the SICU SICU: surgical intensive care unit

Of the 200 surveys administered, 93 healthcare providers working in the surgical ICU completed the survey, yielding a 46.5% response rate. Nurses constituted the largest group of respondents (53%, n=49), followed by resident physicians (23%, n=21), respiratory therapists (18%, n=17), speech pathologists (4%, n=4), and one each of occupational therapists (1%) and nurse practitioners (1%) (Figure 1A).

The majority of respondents (71%, n=66) had less than five years of experience working in a surgical ICU, while 15% (n=14) had 5-14 years of experience, and 14% (n=13) had over 15 years of experience (Figure 1B). Notably, 79% (n=73) reported prior experience working in other hospital settings outside the surgical ICU.

Most healthcare providers (81%, n=75) did not believe that open ICU visiting hours would enhance patient-centered care. Instead, they expressed concerns that open visitation could increase stress on the healthcare team (89%), create physical barriers to patient access (80%), disrupt workflow (90%), and contribute to provider burnout (76%). In contrast, providers perceived that a restricted visitation policy would preserve patient privacy (87%), promote better healing for admitted patients (64%, n=60), reduce provider stress (81%), and improve workflow (95%). A summary of pooled healthcare provider responses is summarized in Table 1.

**Table 1:** Summary of a five-point Likert scale questionnaire assessing healthcare providers' perspectives on open vs. restrictive visitation policy in surgical ICU settings (n=93) ICU: intensive care unit

	Strongly agree	Agree	Neutral	Disagree	Strongly disagree
Open ICU visitation model					
Open ICU visiting hours provide better patient-centered care.	3.2%	9.7%	6.5%	44.1%	36.6%
Open ICU visiting hours will cause increased stress on the healthcare team.	59.8%	29.3%	6.5%	1.1%	3.3%
Open ICU visiting hours can help relieve the stress of patients' families.	1.1%	29%	31.2%	31.2%	7.5%
Open ICU visiting hours will cause more stress upon the patient.	28%	33.3%	24.7%	12.9%	1.1%
Open ICU visiting hours will cause an increase in physical barriers to access the patient.	53.8%	25.8%	7.5%	12.9%	0%
Open ICU visiting hours will cause an interrupted workflow.	62.4%	28%	6.5%	3.2%	0%
Open ICU visiting hours will increase healthcare worker burnout.	60.2%	16.1%	14%	9.7%	0%
Open ICU visiting hours will improve communication between patients' families and healthcare providers.	3.2%	30.1%	30.1%	29%	7.5%
Restrictive ICU visitation model					
Restricted ICU visiting hours help preserve the privacy of patients.	63.4%	23.7%	6.5%	4.3%	2.2%
Restricted ICU visiting hours lead to isolation felt by the patient.	3.2%	24.7%	30.1%	33.3%	8.6%
Restricted ICU visiting hours decrease stress felt by healthcare staff.	47.3%	34.4%	7.5%	7.5%	3.2%
Restricted ICU visiting hours lead to increased communication barriers between patients, their families, and healthcare staff.	3.2%	17.2%	25.8%	43%	10.8%
Restricted ICU visiting hours make performing necessary procedures easier.	67.7%	26.9%	2.2%	1.1%	2.2%
Restricted ICU visiting hours cause a patient to be more relaxed and lead to better healing.	33.3%	31.2%	21.5%	10.8%	3.2%

Of the 14 questions surveyed (Part II of the questionnaire), only two revealed significant differences in responses across provider types. Specifically, nurses were more likely than resident physicians to perceive that open ICU hours would alleviate familial stress (p=0.022). Additionally, nurses and other healthcare providers recognized the potential of open ICU hours to improve communication between patient families and healthcare teams (p<0.05). However, no significant differences were found among providers regarding their perceptions of the impact of open versus restricted visitation on provider stress, workflow, or other factors (p>0.05 for all comparisons).

## Discussion

Utilizing a five-point Likert scale questionnaire, our cross-sectional survey provides insights into healthcare providers' perspectives on open versus restricted visitation policies in a single-institution surgical ICU in the United States. Our study findings reveal a preference among providers for the current restricted visitation policy (four 30-minute visits per day), which they perceive as essential for safeguarding patient privacy, enabling focused care, and fostering a supportive environment for family interactions. While "open visitation" was left open to subjective interpretation due to a lack of standardized definitions in the literature, providers expressed concerns that transitioning to open visitation could negatively impact their work environment without significantly improving patient care.

Lack of standardization in nomenclature and definitions

The lack of a standardized nomenclature for "open" ICU visiting hours complicates the interpretation and comparison of research findings. The term "open" visitation encompasses a wide range of policies, from flexible hours to completely unrestricted access. This variability highlights the diverse approaches institutions adopt, each tailored to their resources and unique circumstances [22]. A review by Ning and Cope, which included 16 studies published between 2013 and 2018, found no consensus on the definition of open visitation in adult ICUs [23]. In an early single-center pilot randomized trial from Italy, Fumagalli et al. defined "unrestrictive" visitation as allowing visits based on patient preference, limited to one visitor at a time, while "restrictive" visitation was limited to two predefined 30-minute visits per day [5]. This variability is echoed in recent randomized studies by Rosa et al. [11] and Yang et al. [8]. In the largest trial on the comparative effectiveness of "flexible family visitation" versus "restrictive family visitation," Rosa et al. defined the flexible model as up to 12 consecutive hours per day for up to two family members who attended a structured information session conducted by trained ICU staff [11,24]. These sessions covered the ICU environment, multidisciplinary work, common ICU treatments, palliative care, infection control practices, delirium prevention, and rehabilitation. Additionally, visitors received educational brochures and access to a website focused on ICU practices and family engagement, aiming to improve cooperation without increasing staff workload [11,24]. In contrast, the restrictive model limited visitation to a maximum of 4.5 hours per day without a mandatory information session [11,24]. Similarly, Yang et al., in a Chinese open-label, parallel randomized controlled trial (RCT) involving 240 patients, defined their restrictive group as allowing one 30-minute visit for up to three visitors, while the flexible group included the same 30-minute visit plus additional visits scheduled based on patient or family needs leveraging a protocol embedded in their "appointment system" [8]. This lack of standardization underscores the need for a framework establishing clear, acceptable definitions for visitation models to enhance the comparability and generalizability of research findings.

Conflicting evidence and the need for further research

The evidence on the efficacy of open visiting hours in ICUs is mixed, with variability in data across high-evidence studies, for example, a prospective single-center study by the ICU Visits Study Group Investigators in Brazil, which enrolled 287 patients in a 31-bed medical-surgical ICU [9]. The authors found that an extended visitation model was associated with significantly lower delirium rates (9.6% vs. 20.5%; adjusted relative risk (aRR): 0.50), shorter delirium/coma duration (1.5 days vs. 3 days; p=0.03), and reduced ICU length of stay (3 days vs. 4 days; p=0.04) compared to a restricted visitation model [9]. However, a subsequent cluster-crossover randomized trial by the same group conducted in over 30 adult ICUs across Brazil found no significant difference in delirium incidence between flexible and restricted visitation policies (18.9% vs. 20.1%; adjusted difference: -1.7% (95% CI: -6.1% to 2.7%); p=0.44) [11]. Conversely, Yang and colleagues reported a lower incidence of delirium in the flexible visitation group compared to the restricted group (5.7% vs. 17.1%; p=0.003) in their cohort of 240 patients [8].

In a pilot randomized trial from Italy, Fumagalli et al. compared "unrestrictive" and restrictive visitation policies in a cohort of 226 patients by alternating the two at their six-bed institutional ICU over a two-year period [5]. The authors found that patients in the unrestrictive group received more frequent daily visits (3.2 vs. 2; p<0.001) which also amounted to be longer on average (2.6 hours vs. 1 hour; p<0.001) [5]. Similarly, Rosa et al. reported a significantly higher mean daily duration of visits in the flexible visitation group (4.8 vs. 1.4 hours; adjusted difference: 3.4 hours (95% CI: 2.8-3.9); p<0.001) [11]. In contrast, Yang et al. reported no significant differences in visitation times per day between the flexible and restricted groups (24.7 minutes vs. 23.9 minutes; p>0.05) but noted higher satisfaction rates (98.6% vs. 92.1%; p=0.011) and a shorter ICU length of stay (6 days vs. 8 days; p=0.041) [8]. No significant differences were observed in hospital-acquired infections (20% vs. 20.7%; p=0.882) or overall hospital stay duration (17 days vs. 19 days; p=0.923) [8]. While some studies suggest the benefits of flexible hours in reducing patient delirium and anxiety [7-9], other research has shown no significant differences in patient or provider outcomes between open and restricted visitation policies [11,23]. Rosa et al. reported no significant differences in ICU-acquired infections (3.7% vs. 4.5%; adjusted difference: -0.8% (95% CI: -2.1% to 1%); p=0.38) or staff burnout (22% vs. 24.9%; adjusted difference: -3.8% (95% CI: -4.8% to 12.5%); p=0.36) [11].

In the United States, studies on ICU visitation policies are relatively scarce. A 2008 survey of 606 US hospitals revealed that most ICUs had restrictive visitation policies, particularly in larger hospitals [21]. The survey also found regional differences: hospitals in the Midwest had the least restrictive policies, while those in the Northeast had the most restrictive [21]. The study noted that ICU nurses in the Northeast expressed concerns about space constraints and conflicts associated with open visitation policies [21], similar to sentiments expressed by providers in our institution. Interestingly, our findings suggest that surgical ICU nurses perceive the potential benefits of open visitation in enhancing communication between healthcare teams and patient families. This nuanced landscape underscores the need for further research on the impact of open visitation on patient outcomes, provider well-being, and family satisfaction in US surgical ICUs.

Future directions

Future research on ICU visitation policies should focus on standardizing the terminology used to describe visitation models, such as "flexible" versus "restrictive," to enable more accurate comparisons across studies. Establishing clear and consistent definitions and cutoffs for these terms is essential to enhance study reproducibility and provide more meaningful interpretations of findings. In the post-COVID-19 era, where visitation practices have been significantly altered, it is also crucial to evaluate how the pandemic has shifted the perspectives of providers, patients, and families towards these policies [25,26]. Research should explore innovative approaches for maintaining family engagement and communication while addressing ongoing infection control needs, particularly in anticipation of future pandemics or public health crises. Including diverse viewpoints from all stakeholders, such as patients and families, is vital for developing comprehensive and adaptable visitation policies that effectively balance patient care, provider well-being, and infection prevention. To overcome implementation challenges associated with open visitation policies, it is important to examine barriers and identify strategies that support successful adoption and sustainability [27]. Key considerations include fostering empathy, utilizing evidence-based practices, applying care models, promoting shared governance, allowing staff discretion, and ensuring appropriate security and family spaces [27].

Limitations

This study has several limitations inherent to a cross-sectional survey design. The survey's intentional ambiguity in defining "open" and "restricted" visitation, while aimed at avoiding response bias, may have introduced subjectivity in interpretation. Additionally, the focus on provider perspectives without input from other key stakeholders, such as patients and families, limits the comprehensiveness of our findings. Future studies should include these perspectives to understand better the impact of visitation policies on all parties involved. Moreover, as our study was conducted before the COVID-19 pandemic, it does not capture the changes in perspectives resulting from the disruption of ICU visitation practices due to social distancing. Future research should explore how the pandemic has shaped attitudes towards visitation and examine new methods for maintaining family connections while adhering to infection control protocols.

## Conclusions

Our study contributes to the ongoing discussion on optimal ICU visitation policies, highlighting the complexity of this issue and the need for further research to fill existing knowledge gaps. By clarifying definitions, incorporating diverse stakeholder perspectives, and considering regional variability, future research can guide the development of evidence-based visitation policies that enhance patient and family well-being while supporting the needs of healthcare providers.

## Figures and Tables

**Figure 2:**
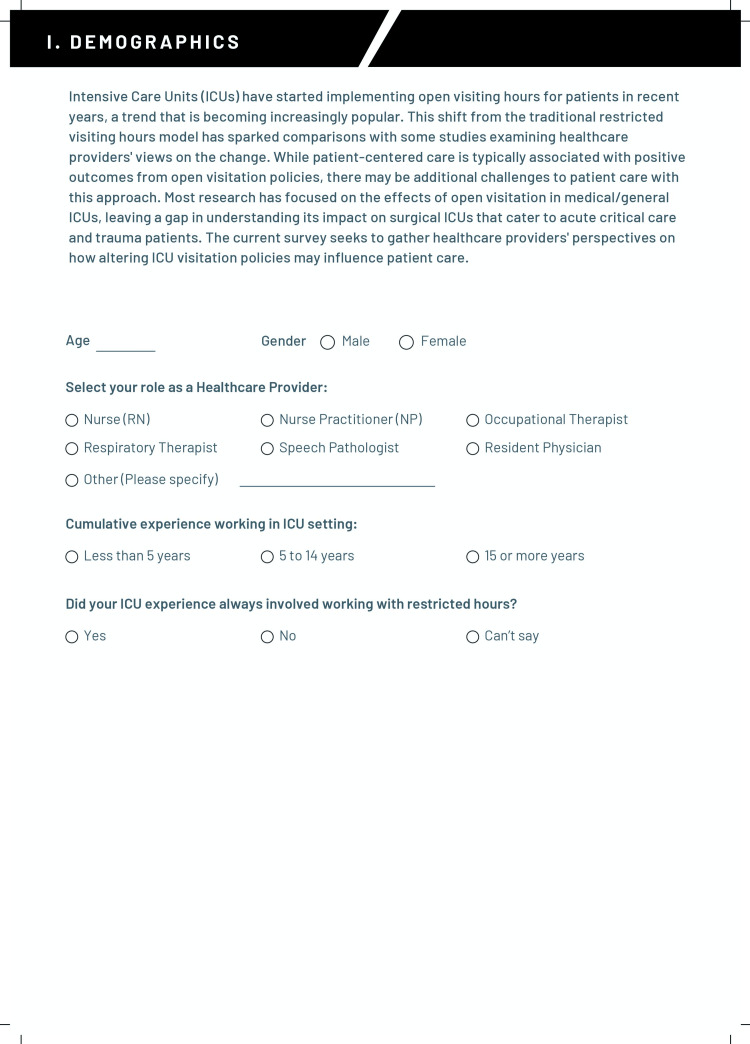
Survey

**Figure 3:**
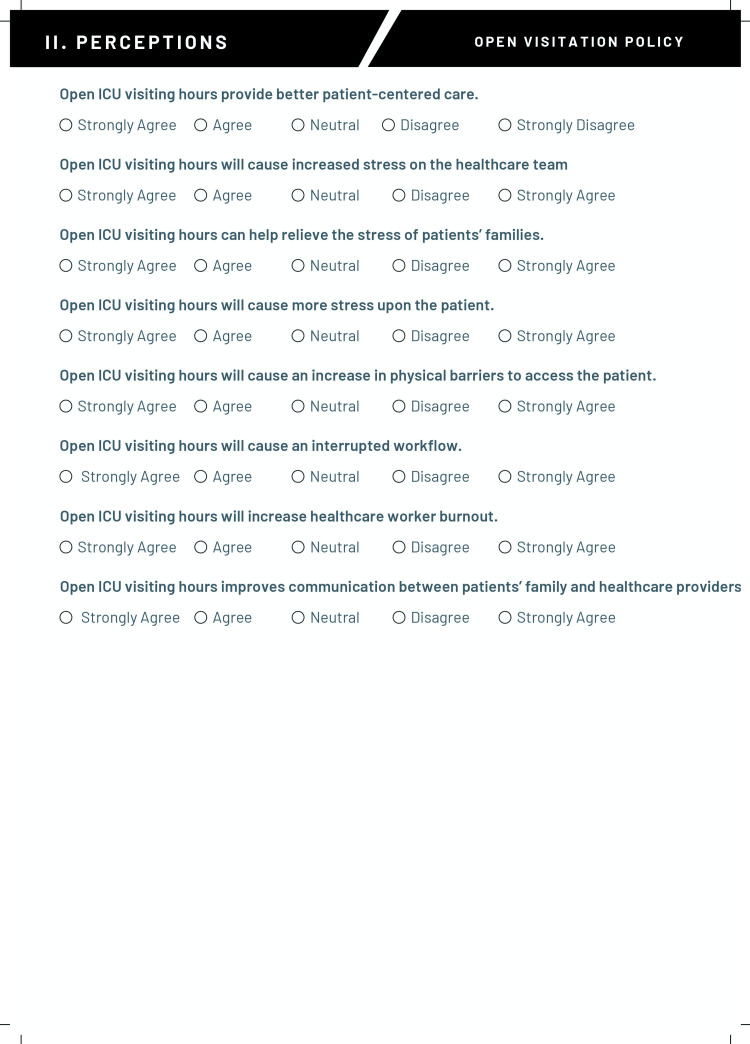
Survey (Contd.)

**Figure 4:**
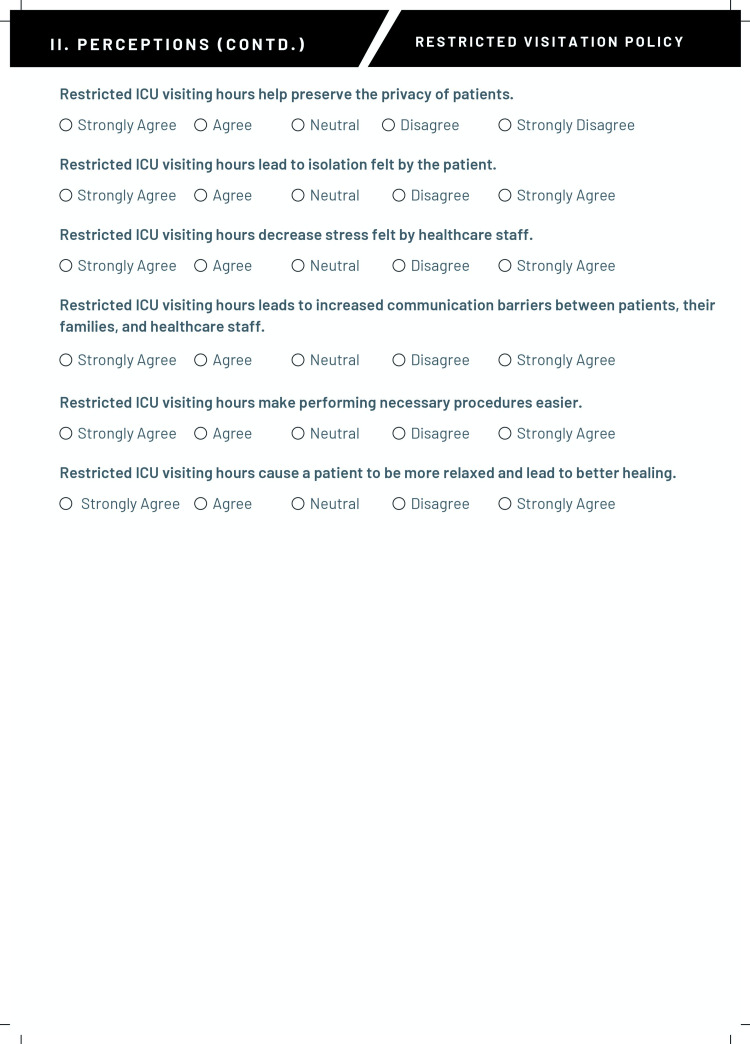
Survey (Contd.)

**Figure 5:**
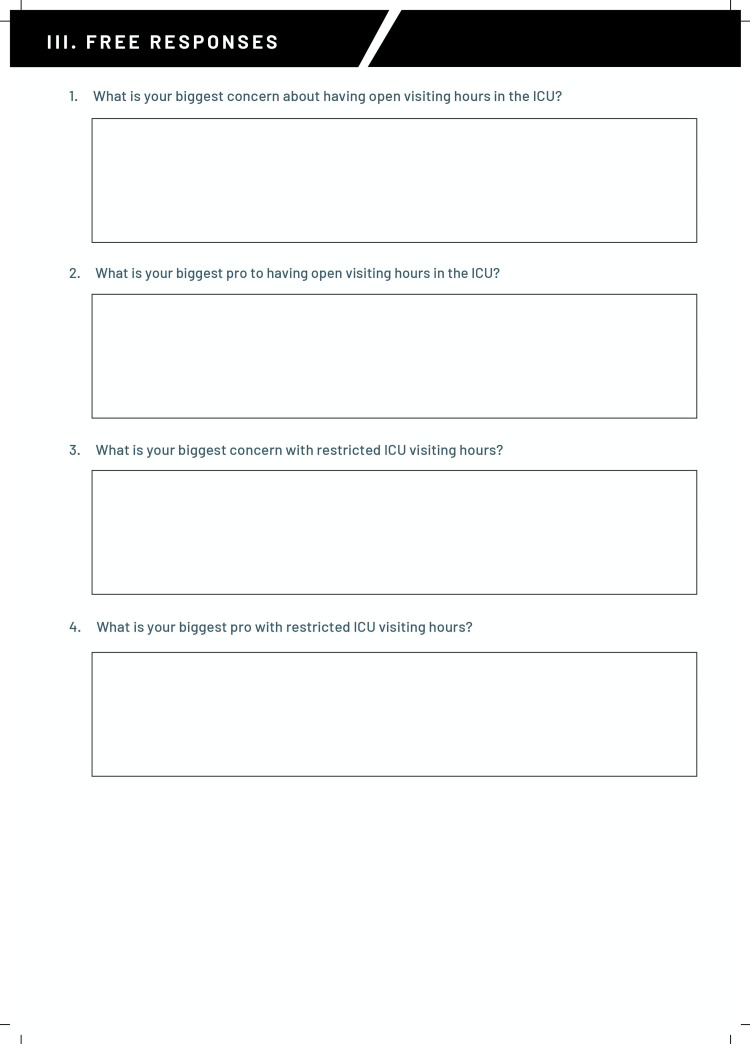
Survey (Contd.)
